# Cumulative incidence and factors associated with unfavorable tuberculosis treatment outcomes among people living with HIV in Liangshan, China: a competing-risk analysis

**DOI:** 10.3389/fpubh.2026.1958022

**Published:** 2026-10-21

**Authors:** Ni Yang, Ting Li, Yunkui Li, Lan Xia

**Affiliations:** Sichuan Center for Disease Control and Prevention, Chengdu, Sichuan, China

**Keywords:** China, competing risks, cumulative incidence, Fine–Gray model, HIV, Liangshan, treatment outcome, tuberculosis

## Abstract

**Background:**

Cure or treatment completion precludes a later unfavorable tuberculosis (TB) treatment outcome within the same treatment episode and should be treated as a competing event. We estimated the cumulative incidence and exploratory associated factors for unfavorable outcomes among people with HIV-associated TB in Liangshan, China.

**Methods:**

Aalen–Johansen cumulative incidence functions and Fine–Gray subdistribution hazard models were used. The 14-parameter full model was treated as exploratory; a prespecified three-parameter Fine–Gray sensitivity model, a cause-specific hazards model, and a proportionality assessment were included. Pointwise 95% confidence intervals for the cumulative incidence functions were obtained using 2,000 individual-level bootstrap resamples.

**Results:**

Among 340 closed treatment episodes, 27 unfavorable outcomes, 303 cure/completion events, and 10 administrative censorings were observed. The unfavorable cumulative incidence was 4.41% (95% CI 2.35–6.77) at 3 months, 6.47% (3.82–9. 12) at 6 months, 7.65% (5.00–10.60) at 9 months, and 7.96% (5.30–10.91) at 12 months. Twenty-two of 27 unfavorable outcomes (81.5%) were records coded as non-TB death; the source field contained no separate TB-related-death category. In the full exploratory model, age was associated with the outcome (adjusted sHR 1.54 per 10 years, 95% CI 1.02–2.32; *p* = 0.039). In the parsimonious model, age (sHR 1.70, 95% CI 1.18–2.43; *p* = 0.004) and tracing versus all other sources (sHR 4.73, 95% CI 1.89–11.86; *p* = 0.001) showed elevated estimates, while inference remained exploratory.

**Conclusion:**

The 12-month unfavorable cumulative incidence was 7.96% (95% CI 5.30–10.91), with only 33 individuals at risk at that landmark. The mortality-dominated composite and sparse categories limit interpretation; the findings describe exploratory associations rather than a validated prediction model.

## 1 Introduction

Tuberculosis (TB) remains the leading cause of death from a single infectious agent. The World Health Organization estimated that 10.7 million people developed TB and 1.23 million died from TB in 2024, including approximately 150,000 deaths among people living with HIV; China remained among the countries contributing most of the global burden (1). HIV increases the risk of progression to active TB and worsens treatment outcomes, making integrated TB/HIV screening, timely treatment, retention, and management of comorbidity central to current guidance (2).

Liangshan Yi Autonomous Prefecture in Sichuan Province is a remote, predominantly ethnic-minority region with a geographically concentrated HIV epidemic and persistent challenges involving access to care, health literacy, adherence, workforce capacity, and socioeconomic vulnerability (3–9). A prefecture-wide analysis of 2,068 people with HIV-associated TB reported a 91.2% anti-TB treatment success rate during 2019–2022 and identified diagnostic and treatment delays as modifiable risk factors (3). A Sichuan cohort also found that deaths clustered early during TB treatment and that older age and markers of advanced disease were important factors associated with death (4).

Prior analysis used Kaplan–Meier (23) estimation and Cox regression for a composite unfavorable outcome, treating cure and treatment completion as censoring events. In the present study, the unfavorable endpoint comprised non-TB death, treatment failure, loss to follow-up, or transfer to multidrug-resistant TB treatment. Cure or treatment completion precluded any later unfavorable outcome within the same treatment episode and was therefore treated as a competing event. Treating a competing event as non-informative censoring changes the estimand and can overestimate the absolute probability of the event of interest (10–18). To our knowledge, the cumulative incidence of this unfavorable endpoint had not previously been estimated in this population while appropriately accounting for cure and treatment completion as competing events. We reanalyzed the original cohort data using cumulative incidence functions and Fine–Gray sub distribution hazards models to estimate absolute risk and examine factors associated with the outcome.

Kajkolahi et al. (19) applied competing-risk methods to an HIV registry cohort with delayed entry and right censoring. Their time origin was estimated HIV infection, with HIV diagnosis defining entry into observation. Our time origin was TB treatment initiation. This distinction highlights the need to separate an administrative extraction boundary from the start of an individual treatment episode and to examine the handling of episodes initiated before that boundary.

## 2 Materials and methods

### 2.1 Study design and setting

We conducted a retrospective cohort analysis of routine patient-level TB case-management records from Liangshan Yi Autonomous Prefecture, Sichuan Province, China. The source workbook contained 521 TB/HIV case records. We included 340 records with a closed treatment episode (status=closed) and excluded 153 episodes still under treatment and 28 re-registered episodes. The administrative observation and censoring window was from 20 October 2024 to 30 June 2026.

### 2.2 Study population and follow-up

Treatment initiation was time zero, and the time scale was elapsed days from the recorded treatment-initiation date. For each closed episode, follow-up ended at the earliest of the recorded treatment-stop date or 30 June 2026. Cure, treatment completion, non-TB death, treatment failure, loss to follow-up, and transfer to multidrug-resistant TB treatment were assigned the recorded treatment-stop date; episodes without an observed event by 30 June 2026 were administratively censored at that date. Two non-positive intervals were set to 0.5 days solely to permit model estimation.

### 2.3 Outcomes and competing events

The event of interest was an unfavorable TB treatment outcome based on the source reason-for-stopping-treatment field: a record coded as non-TB death, treatment failure, loss to follow-up, or transfer to multidrug-resistant TB treatment. The source field contained 22 non-TB-death records and no separate TB-related-death category; therefore, this analysis did not estimate all-cause mortality and did not include a separately coded TB-related death. Transfer to multidrug-resistant TB treatment was classified as unfavorable because it terminated the observed treatment episode and was one of the source system’s unfavorable treatment-outcome categories, not because it was interpreted as biological treatment failure. Cure or treatment completion was treated as a competing event because it precluded a later unfavorable outcome for the same treatment episode. Patients without either event by 30 June 2026 were administratively censored. The six original outcome labels were not merged or redefined.

### 2.4 Covariates and coding

Covariates were mapped one-to-one to the same worksheet fields used in the source manuscript: sex, age, ethnicity, registration category, fixed-dose-combination use, bacteriological result, and patient source. No substitute field or external variable was introduced. Age was continuous and scaled per 10-year increase. Original multicategory levels were retained for descriptive transparency: ethnicity (Yi, Han, and Lisu); FDC use (yes, no, missing); bacteriological result (negative, positive, unavailable); and patient source (direct presentation, tracing, active screening, referral, health examination, recommendation). Original levels were retained in descriptive reporting and the full exploratory model. In the pre-specified parsimonious sensitivity model only, patient source was coded as tracing versus all other sources. Missing FDC values and unavailable bacteriological results were retained as explicit data-completeness categories, not biological exposures. No imputation was performed because the mechanisms of missingness were not documented and may be related to clinical deterioration, early death, diagnostic workflow, or service processes.

The event-of-interest composite consisted of non-TB death, treatment failure, loss to follow-up, or transfer to multidrug-resistant TB treatment. Cure or treatment completion was the competing event, and no event observed by 30 June 2026 was administrative censoring. Reference categories were male, Yi ethnicity, new patient, FDC use = yes, bacteriological result = negative, and patient source = direct presentation. Age was divided by 10 before modeling. The full model used 14 parameters for 27 unfavorable events; category-specific estimates affected by complete or quasi-complete separation were labeled not estimable and were not interpreted.

### 2.5 Statistical analysis

Categorical variables were summarized using counts and percentages; age was summarized using the mean, standard deviation, median, and interquartile range. The Aalen–Johansen estimator was used to calculate cumulative incidence functions (CIFs) for unfavorable and favorable outcomes at 3, 6, 9, and 12 months (11, 15–18). Pointwise 95% confidence intervals were obtained from 2,000 individual-level bootstrap resamples. No formal pairwise or Gray-type comparison of CIFs was performed; CIFs were presented descriptively.

Fine–Gray models were fitted separately for each covariate and in a prespecified full model containing sex, age, ethnicity, registration category, FDC use, bacteriological result, and patient source. A parsimonious sensitivity model contained age, sex, and patient source recoded as tracing versus all other sources; a cause-specific hazards model used the same three covariates. No data-driven variable-selection procedure was used. The full design matrix had rank 14, with no exact collinearity; sparse categories, rather than rank deficiency, were the principal source of instability. The full model was considered exploratory because 27 events supported 14 parameters, whereas inferential emphasis was placed on the parsimonious sensitivity analysis. Results were interpreted as associations with the CIF estimand, not causal effects or validated prediction performance (12–16).

Regression calculations were checked and the proportionality analyses were rerun in Python 3.12.14 with NumPy 2.3.5 and SciPy 1.17.0, using the accompanying script reanalyse_1,958,022.py (functions censor_km, build, fit_fg, and ij_covariance). For the Fine–Gray models, event status was coded as 0 = administrative censoring, 1 = unfavorable outcome, and 2 = cure/treatment completion. The censoring-survival function G was estimated within each analytic cohort using a pooled Kaplan–Meier estimator in which administrative censoring was the event and outcome codes 1 and 2 were censored at their observed times; no censoring strata were used. At each unfavorable-event time t, event-free individuals contributed weight 1 to the sub distribution risk set, whereas individuals with a prior competing event at time Ti remained with inverse-probability-of-censoring weight G(t−)/G(Ti−); individuals with a prior unfavorable event or administrative censoring were no longer at risk. Left limits were used in both numerator and denominator, so outcome events tied with a censoring time were included immediately before the censoring jump at that time. Weights were neither stabilized nor truncated. Non-positive denominators would have triggered an error; none occurred. The minimum censoring-survival denominator and minimum retained competing-event weight were both 0.971834, and the maximum retained competing-event weight was 1.000000. G was re-estimated for each sensitivity-analysis cohort.

Tied unfavorable events were handled with a common Breslow-type denominator; two unfavorable events occurred at day 47. Coefficients were estimated by BFGS optimization of the weighted log pseudo-likelihood with a maximum of 2,000 iterations and up to 10 Newton refinements when needed. Convergence was defined by a maximum absolute score below 1 × 10^−8^ or a relative objective change below 1 × 10^−8^. For the Fine–Gray regression estimates reported in Tables 1 and 2, covariance was obtained from the inverse observed information matrix, treating the estimated censoring weights as fixed; 95% confidence intervals were formed on the log-sHR scale and reported *p* values were two-sided normal-reference Wald tests. This inverse-information covariance is not a robust sandwich estimator and does not incorporate uncertainty from estimating G. Because the weighted objective is not a conventional full likelihood, likelihood-ratio *p* values were not used for the Fine–Gray regression results or multi-degree-of-freedom categorical comparisons.

**Table 1:** Baseline characteristics and observed competing-risk status (*N* = 340).

Characteristic	Category	*n* (%)	Unfavorable, *n* (%)	Cure/completion, *n* (%)
Sex	Male	260 (76.5)	24 (9.2)	229 (88. 1)
	Female	80 (23.5)	3 (3.8)	74 (92.5)
Ethnicity	Yi	332 (97.6)	26 (7.8)	296 (89.2)
	Han	7 (2. 1)	1 (14.3)	6 (85.7)
	Lisu	1 (0.3)	0 (0.0)	1 (100.0)
Registration category	New patient	289 (85.0)	23 (8.0)	260 (90.0)
	Relapse	51 (15.0)	4 (7.8)	43 (84.3)
FDC use	Yes	170 (50.0)	15 (8.8)	152 (89.4)
	No	163 (47.9)	7 (4.3)	149 (91.4)
	Missing	7 (2. 1)	5 (71.4)	2 (28.6)
Bacteriological result	Negative	118 (34.7)	4 (3.4)	109 (92.4)
	Positive	219 (64.4)	20 (9. 1)	194 (88.6)

Characteristic	Category	*n* (%)	Unfavorable, *n* (%)	Cure/completion, *n* (%)
Patient source	Unavailable	3 (0.9)	3 (100.0)	0 (0.0)
	Direct presentation	120 (35.3)	3 (2.5)	114 (95.0)
	Tracing	163 (47.9)	21 (12.9)	135 (82.8)
	Active screening	13 (3.8)	1 (7.7)	12 (92.3)
	Referral	29 (8.5)	2 (6.9)	27 (93. 1)
	Health examination	7 (2. 1)	0 (0.0)	7 (100.0)
Age, years	Recommendation	8 (2.4)	0 (0.0)	8 (100.0)
	Mean (SD)	41.04 (10.89)		
	Median (IQR)	41 (36–47)		

**Table 2:** Cumulative incidence of unfavorable and favorable treatment outcomes with 95% confidence intervals.

Month	Number at risk	Cumulative unfavorable events	CIF unfavorable, % (95% CI)*	CIF cure/completion, % (95% CI)*
3	324	15	4.41 (2.35–6.77)	0.29 (0.00–0.88)
6	268	22	6.47 (3.82–9. 12)	14.71 (11.18–18.53)
9	122	26	7.65 (5.00–10.60)	55.73 (50.47–60.75)
12	33	27	7.96 (5.30–10.91)	81.83 (77.64–85.79)

*95% confidence intervals in Table 4 were obtained using 2,000 individual-level bootstrap resamples with percentile intervals.

The proportional subdistribution-hazards assumption was assessed in the parsimonious Fine–Gray model by adding age×ln(t), sex×ln(t), and tracing×ln(t) interactions simultaneously, yielding a six-parameter model. The joint null hypothesis that all three interaction coefficients were zero was tested with a Wald statistic referenced to a *χ*^2^ distribution with 3 degrees of freedom using an episode-level infinitesimal-jackknife sandwich covariance. The censoring-survival estimate G and the weighted score were re-estimated under each case-weight perturbation, so this covariance incorporated uncertainty from estimating the censoring weights. Variable-specific interaction tests used two-sided normal-reference Wald tests. The same six-parameter interaction model was also evaluated with inverse-information covariance as a variance-sensitivity analysis. No conventional likelihood-ratio test was used for this diagnostic. All tests were two-sided; *p* < 0.05 was considered statistically significant.

## 3 Results

### 3.1 Cohort characteristics and observed outcomes

Among 340 patients, 260 (76.5%) were male, 332 (97.6%) were Yi, and 289 (85.0%) were newly registered. Mean age was 41.04 years (SD 10.89), and median age was 41 years (IQR 36–47). FDC use was recorded as yes in 170 patients (50.0%), no in 163 (47.9%), and missing in 7 (2. 1%). Bacteriological results were positive in 219 (64.4%), negative in 118 (34.7%), and unavailable in 3 (0.9%). Tracing was the most common patient source (163, 47.9%), followed by direct presentation (120, 35.3%). Percentages for outcomes are calculated within each category. Ten patients were censored and are therefore not shown in the two outcome columns. (Table 3).

**Table 3:** Exploratory univariable and full multivariable Fine–Gray regression for the unfavorable outcome.

Variable/category	Unadjusted sHR (95% CI)	*p* value	Adjusted sHR (95% CI)	*P* value
Sex: female vs. male	0.39 (0. 12–1.30)	0. 125	0.46 (0. 14–1.57)	0.217
Age, per 10-year increase	1.55 (1. 11–2. 15)	0.010	1.54 (1.02–2.32)	0.039
Ethnicity: Han vs. Yi	1.94 (0.26–14.32)	0.515	1.03 (0. 11–9.24)	0.980
Ethnicity: Lisu vs. Yi	Not estimable*	_—_	Not estimable*	_—_
Registration: relapse vs. new patient	0.99 (0.34–2.86)	0.986	0.83 (0.22–3.21)	0.792

Variable/category	Unadjusted sHR (95% CI)	*P* value	Adjusted sHR (95% CI)	*P* value
FDC: no vs. yes	0.47 (0. 19–1. 16)	0. 103	0.53 (0. 19–1.48)	0.224
FDC: missing vs. yes	12.51 (4.50–34.79)	<0.001	5.41 (1.48–19.77)	0.011
Bacteriology: positive vs. negative	2.80 (0.96–8. 18)	0.061	2.29 (0.75–7.02)	0. 145
Bacteriology: unavailable vs. negative	121.73 (24.87–595.84)	<0.001	79.77 (11.63–547.26)	<0.001
Source: tracing vs. direct presentation	5.45 (1.63–18.29)	0.006	3. 12 (0.81–11.93)	0.097
Source: active screening vs. direct presentation	3. 19 (0.33–30.67)	0.315	3. 14 (0.32–30.67)	0.326
Source: referral vs. direct presentation	2.81 (0.47–16.79)	0.259	2.23 (0.33–15. 12)	0.413
Source: health examination vs. direct presentation	Not estimable*	_—_	Not estimable*	_—_
Source: recommendation vs. direct presentation	Not estimable*	_—_	Not estimable*	_—_

^*^Not estimable indicates sparse-event separation or an effectively infinite confidence interval. The category was retained for descriptive transparency and was not interpreted as evidence of absence or presence of association. The full multivariable model was exploratory; category-specific p values are Wald-based and do not establish robust clinical effects.

Before the cutoff, 27 patients (7.9%) had an unfavorable outcome and 303 (89. 1%) had the competing outcome of cure or treatment completion; 10 (2.9%) were administratively censored. The unfavorable outcomes comprised 22 source-coded non-TB deaths, 3 transfers to multidrug-resistant TB treatment, 1 treatment failure, and 1 loss to follow-up. Thus, non-TB deaths accounted for 22 of 27 unfavorable outcomes (81.5%), and no record was coded as a separate TB-related death in the supplied outcome field. Cause-of-death classification was taken from routine case-management records and may be subject to information bias.

### 3.2 Cumulative incidence

The CIF of an unfavorable outcome was 4.41% (95% CI 2.35–6.77) at 3 months, 6.47% (3.82–9. 12) at 6 months, 7.65% (5.00–10.60) at 9 months, and 7.96% (5.30–10.91) at 12 months. The corresponding CIFs for cure or treatment completion were 0.29% (0.00–0.88), 14.71% (11.18–18.53), 55.73% (50.47–60.75), and 81.83% (77.64–85.79), respectively (Table 4 and Figure 1). More than half of the 12-month unfavorable CIF had accrued by 3 months (4.41%/7.96% = 55.5%). The increasing occurrence of cure or treatment completion after 6 months is a descriptive pattern consistent with the treatment time scale. The 12-month estimate should be interpreted cautiously because only 33 individuals remained at risk at that landmark.

**Table 4:** Sensitivity analyses for factors associated with the unfavorable outcome.

Model	Variable	Estimate (95% CI)	*p* value
Parsimonious Fine–Gray	Age, per 10-year increase (sHR)	1.70 (1. 18–2.43)	0.004
Parsimonious Fine–Gray	Female vs. male (sHR)	0.45 (0. 13–1.49)	0. 188
Parsimonious Fine–Gray	Tracing vs. all other sources (sHR)	4.73 (1.89–11.86)	<0.001
Cause-specific hazards	Age, per 10-year increase (HR)	1.73 (1.20–2.50)	0.003
Cause-specific hazards	Female vs. male (HR)	0.44 (0. 13–1.47)	0. 183
Cause-specific hazards	Tracing vs. all other sources (HR)	4.62(1.84–11.59)	0.001

^The parsimonious models included age, sex, and tracing versus all other sources.^

**Figure 1:**
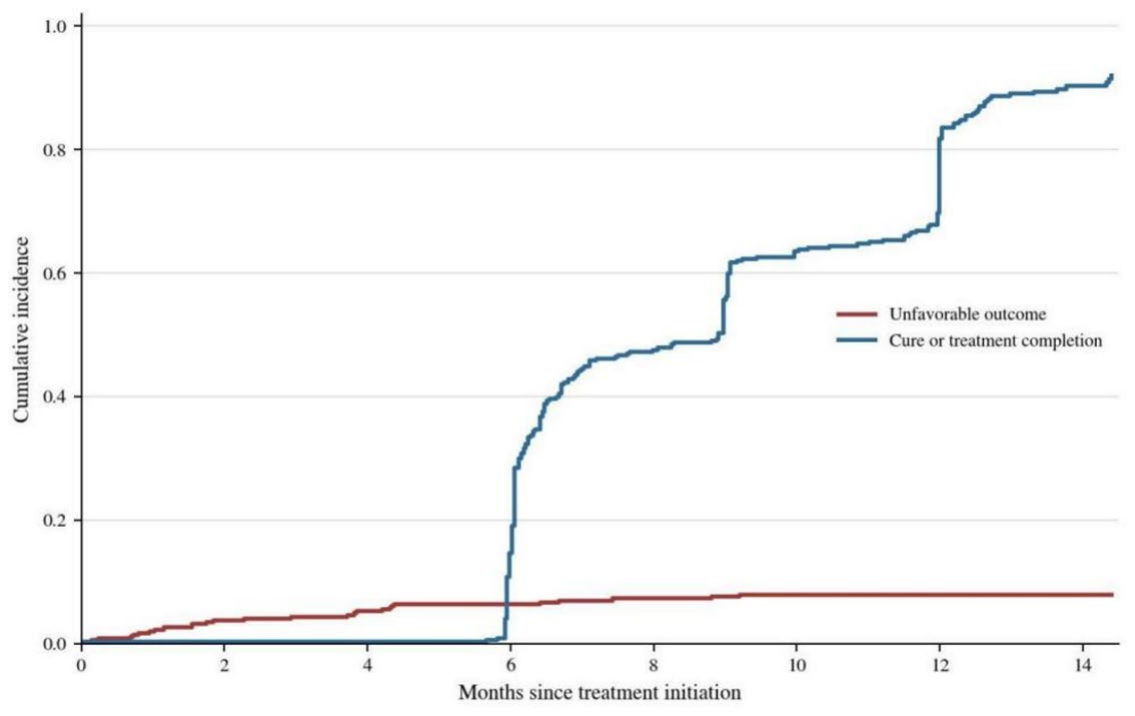
Aalen–Johansen cumulative incidence functions for the unfavorable outcome and the competing favorable outcome of cure or treatment completion.

### 3.3 Univariable and multivariable Fine–Gray models

In univariable analysis, older age was associated with the unfavorable CIF estimand (sHR 1.55 per 10-year increase, 95% CI 1. 11–2. 15; *p* = 0.010). Compared with direct presentation, tracing had an unadjusted sHR of 5.45 (95% CI 1.63–18.29; *p* = 0.006). Estimates for FDC status and bacteriological results were dominated by the very small missing and unavailable categories: five of seven patients with missing FDC status and all three patients with unavailable bacteriological results had unfavorable outcomes. These point estimates and *p* values should be interpreted as sparse-data signals and not as robust evidence of clinical effects. (Table 1).

In the full exploratory multivariable model, each 10-year increase in age was associated with the subdistribution hazard (adjusted sHR 1.54, 95% CI 1.02–2.32; *p* = 0.039). Tracing remained elevated but inconclusive (adjusted sHR 3. 12, 95% CI 0.81–11.93; *p* = 0.097). Missing FDC status (adjusted sHR 5.41, 95% CI 1.48–19.77; *p* = 0.011) and unavailable bacteriological results (adjusted sHR 79.77, 95% CI 11.63–547.26; *p* < 0.001) had extreme point estimates, but their small cell sizes make them highly unstable and unsuitable for quantitative or causal interpretation. Lisu ethnicity, health-examination source, and recommendation source had no unfavorable events and their category-specific sHRs were not estimable. The full model therefore provides exploratory adjusted associations only (Table 1).

### 3.4 Parsimonious models and proportionality assessment

In the parsimonious Fine–Gray model including age, sex, and tracing versus all other sources, age was associated with the unfavorable CIF estimand (sHR 1.70, 95% CI 1.18–2.43; *p* = 0.004), while the tracing estimate was elevated (sHR 4.73, 95% CI 1.89–11.86; *p* = 0.001) but remains exploratory. The cause-specific hazards analysis gave directionally consistent estimates for age (HR 1.73, 95% CI 1.20–2.50; *p* = 0.003) and tracing (HR 4.62, 95% CI 1.84–11.59; *p* = 0.001). (Table 2). The joint log(time) interaction Wald statistic was 9.096 (3 degrees of freedom; *p* = 0.028) using the infinitesimal-jackknife covariance that incorporated estimation of G. Variable-specific interaction *p* values were 0.633 for age, 0.025 for sex, and 0.437 for tracing. With the inverse-information covariance from the same model, the joint statistic was 2.536 (3 degrees of freedom; *p* = 0.469), and the corresponding individual *p* values were 0.707, 0.162, and 0.528. Thus, evidence of non-proportionality, principally involving sex, was sensitive to variance estimation. With only three unfavorable events among women, these asymptotic tests were unstable; proportionality could not be regarded as established. The constant sHRs in Table 2 should be interpreted as working-model summaries over follow-up, particularly for sex, and these diagnostics do not validate the full exploratory model Figure 2.

**Figure 2:**
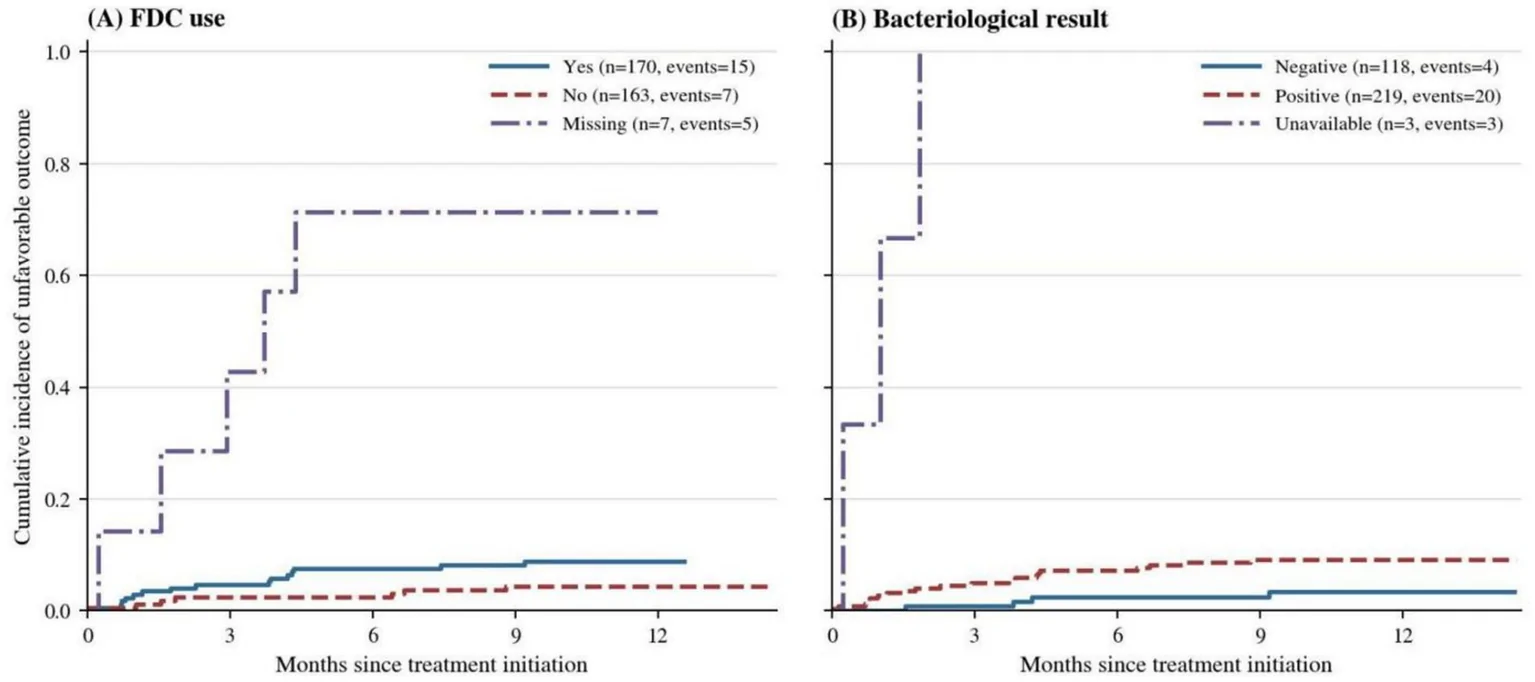
Aalen–Johansen cumulative incidence functions for the unfavorable outcome according to **(A)** the three original fixed-dose-combination (FDC) categories and **(B)** the three original bacteriological-result categories. Categories were not collapsed; the missing FDC and unavailable bacteriology groups were sparse and should be interpreted cautiously.

## 4 Discussion

This competing-risk re-analysis produced three principal findings. First, the 12-month cumulative incidence of an unfavorable outcome was 7.96% (95% CI 5.30–10.91) when cure and treatment completion were treated as competing events. Second, older age showed the most consistent exploratory adjusted association across the primary and sensitivity analyses. Third, tracing status and incomplete FDC or bacteriological documentation were associated with elevated estimates in selected analyses, although sparse cells, possible reverse causation, and limited events make these findings unstable. These results are exploratory associations and should not be interpreted as a validated prediction model.

The difference from the Kaplan–Meier framing concerns the estimand. When cure or treatment completion is treated as non-informative censoring, Kaplan–Meier estimation redistributes the remaining probability of those competing events among individuals who remain at risk (20). This can overestimate the probability of an event that cannot occur after treatment completion. The CIF instead represents the observed probability of the unfavorable event in the presence of the favorable competing outcome (10–18). In this cohort, the 12-month unfavorable CIF was 7.96% (95% CI 5.30–10.91), but only 33 individuals remained at risk and the cohort was geographically restricted; the estimate should not be presented as directly sufficient for service planning.

The earlier Liangshan study reported a crude 2019–2022 treatment-success proportion of 91.2% (3). That proportion is not directly comparable with the present 12-month CIF of cure or treatment completion (81.83%) because the studies differ in denominator, study period, follow-up duration, outcome definition, and treatment of censoring. We therefore no longer describe the two results as consistent. In the present data, 22 of 27 unfavorable outcomes were source-coded non-TB deaths, so the composite is dominated by mortality rather than representing all unfavorable treatment outcomes in a balanced way. Any implication for broad TB/HIV care is a hypothesis for future evaluation, not a demonstrated effect of this analysis.

Age showed the most consistent exploratory adjusted association with the unfavorable CIF estimand. In the parsimonious Fine– Gray model, the sHR was 1.70 per 10-year increase (95% CI 1.18–2.43; *p* = 0.004), and the corresponding cause-specific hazard ratio was 1.73 (95% CI 1.20–2.50; *p* = 0.003). Reduced physiological reserve, comorbidity, polypharmacy, delayed presentation, or treatment toxicity are plausible hypotheses, but these mechanisms were not measured. Age should therefore not be described as an independent predictor or used as a standalone clinical prediction rule.

Patients identified through tracing had an elevated univariable sHR and an elevated but inconclusive full-model estimate (adjusted sHR 3. 12, 95% CI 0.81–11.93; *p* = 0.097). In the parsimonious sensitivity model, the estimate was higher, but it remains an exploratory association. The analysis did not calculate or report a stratified crude CIF by source, so the result is not described as a higher crude cumulative incidence. Possible explanations involving previous disengagement, delayed presentation, disease severity, mobility, stigma, or structural barriers were not measured and are presented only as hypotheses. Liangshan’s comprehensive HIV strategy has strengthened testing, linkage, and grassroots follow-up (6), while qualitative work has documented persistent adherence challenges (9). Tracing status may justify multidimensional assessment and retention support, not a causal interpretation.

The large coefficients for missing FDC status and unavailable bacteriological results should not be treated as clinical characteristics or causal effects. The source system did not record why these fields were missing. Incomplete documentation, diagnostic workflow, severe illness, early death, treatment interruption, and service processes may all contribute. Reverse causation is possible if early deterioration or death prevented completion of the relevant fields. Because five of seven patients with missing FDC status and all three with unavailable bacteriology had unfavorable outcomes, sparse-data bias and information bias are unavoidable. The appropriate programmatic response is to improve documentation and investigate missingness mechanisms, not to infer treatment effects. FDCs remain operationally useful and are supported by TB treatment guidance (2, 21, 22).

### 4.1 Strengths and limitations

Strengths include explicit definition of the CIF estimand, transparent handling of cure and treatment completion as competing events, reporting of uncertainty for absolute CIFs, and complementary parsimonious, cause-specific, proportionality, and pre-window sensitivity analyses. The original source categories are shown for descriptive transparency, but their retention is not presented as an unqualified statistical strength.

The study has important limitations. Only 27 unfavorable outcomes occurred, while the full multivariable model contained 14 parameters; overfitting, wide confidence intervals, sparse-event separation, and unstable *p* values were therefore expected. The composite outcome was clinically heterogeneous and dominated by 22 source-coded non-TB deaths; routine cause-of-death classification may introduce information bias, and no separately coded TB-related death was available. The retrospective secondary-data design may include errors in event dates and outcome classification, residual confounding, selection bias related to cohort assembly, and dependence between documentation quality and early deterioration. The analysis assumes independent censoring and has insufficient power for outcome-specific analyses. The proportionality diagnostic was restricted to the estimable parsimonious model. The sandwich-based interaction diagnostic suggested non-proportionality involving sex, whereas the inverse-information diagnostic did not; with only three unfavorable events among women, this disagreement and the limited event count constrain interpretation of constant sHRs. Proportionality was not assessed reliably in the sparse 14-parameter model. The surveillance extract lacked CD4 count, HIV viral load, ART timing and adherence, body mass index, anemia, other opportunistic infections, TB site and severity, drug susceptibility, adverse reactions, and socioeconomic measures. Finally, the almost entirely Yi cohort from one prefecture limits generalizability. All multivariable findings are exploratory, cannot establish causality, and do not demonstrate predictive performance.

### 4.2 Implications

Future studies should link TB and HIV databases, prospectively define competing outcomes, improve completeness of FDC and bacteriological fields, and prespecify a parsimonious model supported by more events. Larger datasets should assess cause-specific and subdistribution hazards together, evaluate delayed entry and independent censoring, and verify proportional subdistribution hazards in an adequately powered model. Multiple imputation should not be assumed to solve the present missingness problem: investigators should first study the missingness mechanism and, only when assumptions are plausible, combine imputation with sensitivity analyses for data potentially missing not at random. Prediction models should be considered only after adequate sample size, discrimination, calibration, and external validation are available.

## 5 Conclusion

In this cohort of 340 people with HIV-associated TB in Liangshan, the 12-month cumulative incidence of an unfavorable treatment outcome was 7.96% (95% CI 5.30–10.91) when cure and treatment completion were treated as competing events. Only 33 individuals remained at risk at 12 months. Older age showed the most consistent exploratory association across the primary and sensitivity analyses, whereas tracing status and incomplete FDC or bacteriological documentation were affected by sparse data, possible reverse causation, and residual confounding. Because 22 of 27 unfavorable outcomes were source-coded non-TB deaths, the composite estimate primarily reflects mortality. The findings support better TB/HIV documentation and follow-up, but larger linked studies are needed before causal or clinical prediction claims can be made.

## Data Availability

The raw data can be made available by the authors upon reasonable request.
